# From challenge-skills balance to wellbeing in sports: the role of team identification and flow

**DOI:** 10.3389/fpsyg.2026.1863570

**Published:** 2026-06-22

**Authors:** Manuel Teresi, Alessandra Fermani, Ilaria Giovannelli, Mara Marini, Stefano Livi, Attilio Di Crescenzo, Stefano Pagliaro

**Affiliations:** 1 Department of Education, Cultural Heritage and Tourism, University of Macerata, Macerata, Italy; 2 Department of Psychology, University of Chieti-Pescara, Chieti, Italy; 3 Department of Psychology of Developmental and Socialization Processes, Sapienza University of Rome, Rome, Italy; 4 Department of Neuroscience, Imaging and Clinical Sciences, University of Chieti-Pescara, Chieti, Italy

**Keywords:** challenge-skills balance, flow, psychological wellbeing, sport psychology, team identification

## Abstract

This study investigated the psychological mechanisms linking challenge-skills balance to psychological wellbeing among team-sport athletes, considering team identification and flow experience as the fundamental drivers of this relationship. A sample of 281 team-sport athletes completed an online survey comprising measures of challenge-skill balance, team identification, flow, and wellbeing. Results supported the hypothesized model: Athletes who perceived their competencies as congruent with competitive demands reported stronger team identification, which in turn was related to deeper flow experiences. Flow was then associated with wellbeing, mediating the effect of team identification. These findings highlight the interconnected role of cognitive resources, social identity processes, and optimal experiential states in sustaining athletes’ psychological wellbeing.

## 1 Introduction

Promoting psychological wellbeing among athletes has become a priority in sport psychology, reflecting a shift from deficit-oriented models toward a positive, flourishing conception of mental health (Lundqvist, 2011; Ryan and Deci, 2001). Within this framework, wellbeing encompasses positive affect, vitality, purpose, and personal growth—not merely the absence of distress (Keyes, 2002; Seligman, 2011). The Job Demands–Resources (JD-R) model (Bakker and Demerouti, 2017) provides a useful theoretical lens, positing that wellbeing emerges when personal and contextual resources buffer demands and sustain engagement. In the sport domain, the perception of the challenge-skill balance—the degree to which athletes perceive situational demands as congruent with their skill level, typically assessed via self-report items tapping perceived competence relative to task demands (Jackson and Marsh, 1996)—represents one such key resource (Csikszentmihalyi, 1990; Swann et al., 2018). When congruence is perceived, athletes experience mastery and efficacy; imbalances in either direction produce anxiety or boredom detrimental to performance and wellbeing (Nakamura and Csikszentmihalyi, 2002).

Yet challenge-skills balance does not operate in isolation. Whereas the JD-R framework explains how challenge–skills balance may function as a personal resource sustaining motivation and wellbeing, the Social Identity Approach highlights the relational processes through which athletes derive belongingness and psychological meaning from team membership (Haslam et al., 2020). Flow Theory, in turn, explains how these cognitive and social resources may culminate in optimal experiential states characterized by absorption and intrinsic enjoyment. According to the Social Identity Approach to Sport (Haslam et al., 2020), team membership provides athletes with a source of collective identity that shapes motivation, resilience, and wellbeing (Bruner et al., 2014; Rees et al., 2015); in this study, we operationalized it as the degree to which athletes cognitively define themselves in terms of their team membership (Mael and Ashforth, 1992). Self-Determination Theory (SDT; Deci and Ryan, 2000) further suggests that competence and relatedness needs are mutually reinforcing: feeling effective within a team context simultaneously strengthens psychological belonging, thereby increasing team identification. Team identification, in turn, may facilitate the experience of flow, that is, the state of complete absorption in an intrinsically rewarding activity characterized by loss of self-consciousness and a sense of control (Csikszentmihalyi, 1990), typically captured through self-report measures of absorption, enjoyment, and intrinsic motivation (Bakker, 2008). Although flow is experienced at the individual level, it may also be contextually embedded and socially facilitated in interdependent settings such as team sports.

In fact, belonging to a cohesive team reduces self-referential processing and evaluative concerns, freeing cognitive resources for deep task involvement (Eys et al., 2009; Swann et al., 2012). Together, these frameworks complement each other in a non-redundant way: JD-R explains why challenge-skill congruence matters for wellbeing; the Social Identity Approach explains how team membership amplifies this resource effect via identification (Rees et al., 2015); and Flow Theory explains the experiential mechanism (i.e., optimal absorption) through which identification translates into sustained wellbeing (Nakamura and Csikszentmihalyi, 2002). This sequential logic–from resource appraisal to social identification, experiential absorption, and ultimately wellbeing–defines the specific ordering of the serial mediation model tested here. Once attained, flow contributes to wellbeing through both hedonic (immediate enjoyment) and eudaimonic (long-term vitality and purpose) pathways (Jackson et al., 2001; Waterman, 1993). Although previous sport psychology research has separately linked challenge–skills balance, team identification, flow, and wellbeing, these relationships have rarely been integrated within a single theoretically coherent model (Swann et al., 2018; Rees et al., 2015). Existing studies have predominantly examined dyadic associations, such as the link between flow and performance (Jackson et al., 2001; Swann et al., 2012) or between team identification and wellbeing (Bruner et al., 2014; Rees et al., 2015), leaving unclear how cognitive appraisals of competence, social identity processes, and optimal experiential states jointly contribute to athlete flourishing in team sport contexts. Moreover, although flow theory has traditionally emphasized individual experiential processes (Nakamura and Csikszentmihalyi, 2002), less attention has been devoted to the social and relational conditions that may facilitate flow experiences within interdependent sport environments (Zumeta et al., 2016), to the best of our knowledge. Similarly, research grounded in the Social Identity Approach to Sport has extensively documented the benefits of team identification for motivation and resilience (Haslam et al., 2020; Rees et al., 2015), yet its role in fostering optimal psychological states such as flow remains underexplored.

The present study builds on and extends Marini et al. (2025) model originally tested in organizational settings by examining whether a similar sequential motivational pathway operates in team sports. By integrating Flow Theory (Csikszentmihalyi, 1990), the Social Identity Approach (Haslam et al., 2020), and the JD-R framework (Bakker and Demerouti, 2017), the present research proposes that athletes’ perceptions of challenge–skills balance may enhance wellbeing not only directly, but also indirectly by strengthening identification with the team and facilitating flow experiences. Drawing on SDT and the Social Identity Approach, we hypothesized that (H1) challenge-skill balance would be positively associated with team identification, as feeling competent within a team context reinforces belonging and collective identity; (H2) Identification with the team would be positively associated with flow, as the shared identity attenuates evaluative concerns and frees up cognitive resources for deep absorption in the task; (H3) Flow would be positively associated with psychological wellbeing, reflecting its contribution to both emotional and personal fulfillment; and (H4) team identification and flow would serially mediate the challenge-skill balance–wellbeing relationship, representing the motivational chain linking cognitive resources to optimal experience.

## 2 Materials and methods

### 2.1 Participants and procedure

A Monte Carlo power analysis (Schoemann et al., 2017) indicated that a minimum sample size of *N* = 110 was needed to achieve a power of 0.80 for the serial indirect effect (α = 0.05); a larger sample size was targeted for greater stability. This simulation-based approach relied on correlation coefficients and standard deviations derived from Marini et al. (2025), who tested an analogous sequential motivational model in organizational contexts using the same psychometric instruments.

Participants were recruited via convenience snowball sampling using an online survey (Qualtrics). The survey link was initially distributed within sport science courses at two Italian universities; these initial respondents were then invited to disseminate the questionnaire within their networks, specifically targeting individuals who met the inclusion criteria. Eligibility required being at least 18 years old and an active team-sport athlete. The survey took approximately 15 min to complete. A total of 434 individuals accessed the survey link. To ensure data quality and the reliability of the findings, a complete-case analysis approach was adopted: only questionnaires with 100% completion of the primary study variables were retained, while incomplete or partially filled responses were excluded. The final sample comprised 281 participants (*M* age = 25.65, *SD* = 8.22), of whom 57.8% were male, 41.8% female, and 0.3% non-binary. The majority played football (45%), volleyball (28.2%), or basketball (11.4%). Participants trained an average of 3.4 days/week (*SD* = 0.65), with sessions of 110 min (*SD* = 30.16). Participation was voluntary and anonymous; all participants provided informed consent. The study was approved by the Ethics Committee of the University of Cagliari (Protocol No. 0264430) and adhered to the Declaration of Helsinki.

### 2.2 Measures

To mitigate common method variance, response scales were intentionally varied across instruments, and the items within each scale were randomly ordered. As an additional check for common method bias, Harman’s single-factor test was performed by entering all study items into an exploratory factor analysis with a single factor forced. The single factor accounted for 35.69% of the total variance, well below the 50% threshold, providing no evidence of pervasive common method bias (Podsakoff et al., 2003). challenge-skills balance was assessed with four items from the Flow State Scale (Jackson and Marsh, 1996), adapted for sport (e.g., “My abilities are adequate for the high complexity of the situation”; 5-point scale; α = 0.85). Team identification was measured with the six-item organizational identification scale (Mael and Ashforth, 1992) in its Italian adaptation (Manuti and Bosco, 2012), contextualized to sport team context (e.g., “When someone criticizes my team, I feel it as a personal insult”; 6-point scale; α = 0.71). Flow was assessed using the Work-related Flow Inventory (WOLF; Bakker, 2008), validated in Italian and adapted for sport (Zito et al., 2015, 2018). The WOLF was preferred over sport-specific flow measures (e.g., the Flow State Scale-2; Jackson and Eklund, 2002) to preserve the operational independence between the flow mediator and the challenge-skills balance predictor, given that the latter was assessed using items drawn from the FSS-2 challenge-skill balance subscale. This choice is supported by the validation of the scale in sport contexts by Zito et al. (2018), which demonstrated adequate factorial validity and reliability in performance settings. The 13-item unidimensional scale captures absorption, enjoyment, and intrinsic motivation (5-point scale, α = 0.93). Psychological wellbeing was measured with the five-item WHO-5 Wellbeing Index (Topp et al., 2015), capturing positive mood, vitality, and general interest over the previous 2 weeks (5-point scale, α = 0.85).

### 2.3 Data analysis

Analyses were conducted in IBM SPSS Statistics 28. The serial mediation model was tested using the PROCESS Macro (Model 6; Hayes, 2017), including age, gender, training days, session duration, and team size as covariates. Indirect effects were estimated with 5,000-resample bootstrapping and 95% bias-corrected confidence intervals.

## 3 Results

Means, standard deviations, and zero-order Pearson’s correlations are presented in Table 1. All variables were positively and significantly intercorrelated, consistent with H1–H3.

**Table 1:** Means, standard deviations, and zero-order Pearson correlations among study variables.

Variable	*M* (*SD*)	1	2	3
1. Challenge-skills balance	3.73 (0.74)	–		
2. Team identification	4.26 (0.84)	0.27***	–	
3. Perceived flow	4.25 (0.65)	0.36***	0.35***	–
4. Psychological wellbeing	3.78 (0.73)	0.37***	0.20**	0.60***

** *p* < 0.01; *** *p* < 0.001.

**Figure 1:**
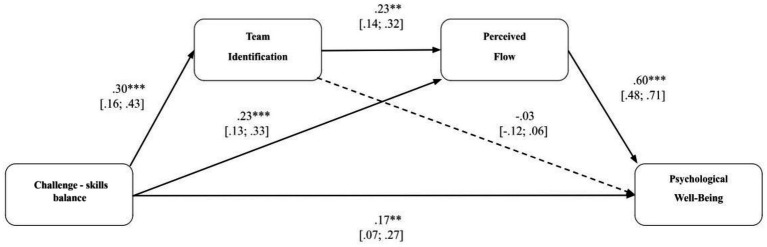
Serial mediation model of the effect of challenge-skills balance on psychological wellbeing through team identification and flow. ** *p* < 0.01; *** *p* < 0.001.

The serial mediation model was significant overall [*R*^2^ = 0.41, *F*(8, 266) = 22.84, *p* < 0.001]. The challenge-skills balance positively predicted team identification [*b* = 0.30, 95% CI: 0.16, 0.43], supporting H1. Team identification positively predicted flow [*b* = 0.23, 95% CI: 0.14, 0.32], supporting H2. Flow strongly predicted psychological wellbeing [*b* = 0.60, 95% CI: 0.48, 0.71], supporting H3. The serial indirect effect of challenge-skills balance on wellbeing through team identification, then flow, was significant [*b* = 0.04, 95% CI: 0.02, 0.07], supporting H4. The direct effect of challenge-skills balance on Wellbeing also remained significant [*b* = 0.17, 95% CI: 0.07, 0.27], indicating partial mediation. Among covariates, gender significantly predicted both team identification (female athletes higher) and wellbeing (male athletes slightly higher). These results are depicted in Figure 1.

## 4 Discussion

This study examined the sequential pathway linking challenge-skills balance to psychological wellbeing via team identification and flow in team-sport athletes. Findings confirmed our hypotheses and contribute to sport psychology knowledge by demonstrating, for the first time in a team sport context, that challenge-skill balance, team identification, flow, and wellbeing operate as an integrated sequential motivational chain, rather than as isolated constructs or simple dyadic associations.

Consistent with H1, perceiving one’s skills as adequate to meet competitive demands was positively associated with team identification. Feeling like an effective contributor to collective goals appears to strengthen athletes’ internalization of the team as part of their self-concept (Tajfel and Turner, 1979). This extends prior work on collective efficacy (Bandura, 1997; Fransen et al., 2014) by showing that individually perceived competence—not only group-level beliefs—can catalyze social identification in sport.

Supporting H2, team identification was positively associated with flow. When athletes feel psychologically anchored within a cohesive team, the social environment buffers evaluative concerns, reduces self-referential processing, and creates the psychological safety necessary for deep task absorption (Eys et al., 2009; Zumeta et al., 2016). This identifies flow facilitation as a specific pathway through which team identification benefits individual athletes. It is worth noting that this association may be specific to team sport contexts; in individual sports, where shared identity is less structurally embedded, the link between identification and flow may be weaker or operate through different mechanisms (e.g., Swann et al., 2012). Consistent with H3, flow was positively related to psychological wellbeing, operating through both hedonic (immediate positive affect) and eudaimonic (long-term vitality and purpose) pathways (Waterman, 1993), replicating prior sport findings (Hodge et al., 2009; Swann et al., 2012). The persistence of a significant direct effect of challenge-skills balance on wellbeing suggests that the team identification and flow pathway does not fully account for this relationship, pointing to additional psychological mechanisms that have to be explored in *ad hoc* research. From a self-determination theory perspective (Deci and Ryan, 2000), challenge-skill congruence may directly satisfy basic psychological needs for competence and autonomy, independently of social identification processes. Alternatively, individual-level coping strategies or emotion regulation mechanisms may constitute additional pathways not captured by the present model.

From an applied standpoint, these results suggest that sports environments fostering both competence development and collective belonging represent the most effective pathway toward athlete flourishing. Practitioners should design progressively demanding training activities tailored to athletes’ competitive level and skill development to maintain an optimal challenge–skill balance. In highly interdependent sports such as volleyball or basketball, interventions fostering communication, role clarity, and collective goal-setting may be particularly effective in strengthening team identification and facilitating shared flow experiences. Conversely, in sports characterized by greater tactical specialization, coaches may need to balance collective cohesion with individualized competence support. Several limitations should be noted. First, the cross-sectional design precludes causal inferences; although the serial mediation model was theoretically grounded and statistically supported, the directionality of the proposed sequential pathway—from challenge-skills balance through team identification to flow and wellbeing—cannot be established from cross-sectional data alone. This is an important caveat: while the theoretical ordering of variables is grounded in prior work (Marini et al., 2025) and supported by the pattern of statistical results, the correlational nature of the data means that reverse or alternative causal pathways cannot be ruled out. Longitudinal designs tracking athletes across a competitive season and experience sampling methodology, which captures flow in its naturally episodic form, would provide stronger evidence for the hypothesized causal sequence. Second, reliance on self-report raises common method variance concerns; although response scales were intentionally varied across instruments to partially mitigate this issue, future studies should incorporate multi-source data or physiological indicators of flow and wellbeing. Third, the convenience sample drawn from Italian university sport sciences students, with a predominance of football players, limits generalizability across competitive levels, sport types, and cultural contexts. In particular, the predominance of football and volleyball players means that sport-specific dynamics, such as differences in team interdependence, role structures, and competitive pressure, may influence the relationships examined here in ways not captured by the present study.

Future research should employ stratified sampling strategies across different competitive levels (amateur, semi-professional, elite) and cultural backgrounds to test whether the proposed model holds with equal strength across the performance spectrum.

Despite these limitations, the present findings offer a theoretically grounded and empirically supported account of how individual cognitive resources, social identity processes, and optimal experiential states jointly sustain wellbeing in competitive team sport.

## Data Availability

The raw data supporting the conclusions of this article will be made available by the authors, without undue reservation.
